# Structural and Electronic Stabilization Tuning of Al_6_N_6_ Clusters via Hydrogenation: A Theory Study of Al_6_N_6_H_8_

**DOI:** 10.3390/molecules31030495

**Published:** 2026-01-31

**Authors:** Peng-Fei Li, Yang Yang, Shu-Juan Gao

**Affiliations:** 1 Institute of Technology, Shanxi Open University, Taiyuan 030027, China; 2 The Key Laboratory of the Materials for Energy Conversion and Storage of Shanxi Province, Institute of Molecular Science, Shanxi University, 92 Wucheng Road, Taiyuan 030006, China; yangyang9@sxu.edu.cn; 3 Department of Chemical and Materials Engineering, Lyuliang University, Lvliang 033001, China

**Keywords:** AlNH cluster, global minimum, HOMO-LUMO gap, theoretical chemistry

## Abstract

Investigating aluminum nitride (AlN) clusters is essential for understanding the properties of bulk AlN materials. The incorporation of hydrogen into AlN clusters represents an effective strategy for structural modification and for tuning their physicochemical properties. In this work, we conducted density functional theory (DFT) calculations on the dynamically stable global-minimum (GM) structure of Al_6_N_6_H_8_. Compared to the precursor Al_6_N_6_ cluster, the incorporation of eight hydrogen atoms achieves coordination saturation of all aluminum and nitrogen atoms, inducing a structural transformation from a hexagonal prism with *D*_3*d*_ symmetry to a cuboid structure with *D*_2*h*_ symmetry. The HOMO–LUMO gap of the Al_6_N_6_H_8_ cluster is increased by 1.85 eV compared to that of Al_6_N_6_, indicating a remarkable enhancement in stability. Chemical bonding and natural bond orbital (NBO) charge analyses reveal that the Al–N, Al–H, and N–H bonds are predominantly covalent single bonds, with a degree of ionicity arising from electronegativity differences. The hydrogen atoms bonded to Al and N can be substituted with a series of other atoms or functional groups, thereby further tuning the structures and properties of the clusters. To facilitate future experimental characterization, the infrared spectrum of Al_6_N_6_H_8_ was calculated, which shows an overall blue shift in the Al–N bond’s bending and stretching vibrations compared to those in the Al_6_N_6_ cluster.

## 1. Introduction

Aluminum nitride (AlN), an important wide bandgap semiconductor material, typically crystallizes in the hexagonal wurtzite structure. It possesses excellent thermal conductivity, a low thermal-inflate coefficient, high chemical stability, and favorable dielectric properties, exhibiting broad application prospects in fields such as optoelectronic devices [1,2,3,4,5,6]. Aluminum nitride clusters, serving as an intermediate system bridging the atomic scale and bulk materials, have become a focus of theoretical research concerning their structures, stability rules, and growth mechanisms [7,8,9,10,11,12,13,14,15,16,17,18,19,20]. Chang et al. [10] employed density functional theory (DFT) to investigate the structures, energies, and vibrational frequencies of (AlN)_x_ (x = 1, 2, 4, 6, 12) clusters, finding that cage-like structures gradually become more stable than planar structures as their size increases, with (AlN)_12_ exhibiting high symmetry and stability. Recently, Xu et al. [13], based on Fukui function analysis and transition-state searches, studied the growth mechanism of Al*_n_*N*_n_* (*n* = 2–9) clusters, proposing a transition from planar to three-dimensional cage-like structures during their growth. Costales et al. [7] utilized global optimization methods to investigate (AlN)*_n_* (*n* = 1–100) clusters, revealing that their structural evolution with size can be divided into three stages: Small-sized clusters (*n* = 2–5) are dominated by planar ring structures. Medium-sized clusters (*n* = 6–40) show competition between stacked rings and globular-like empty cages, and large-sized clusters (*n* > 40) develop interior atoms and exhibit a competition between tetrahedral- and octahedral-like features. This study also revealed that clusters with sizes of *n* = 6 and *n* = 12 are particularly stable in terms of energy, displaying “magic number” characteristics. These works lay a crucial foundation for understanding the stability and growth patterns of aluminum nitride clusters. As the smallest Al*_n_*N*_n_*-type three-dimensional aluminum nitride cluster, Al_6_N_6_ holds significant value for further investigation.

However, despite considerable research elucidating the structural and stability rules of aluminum nitride clusters, theoretical studies on ternary Al-N-H clusters in the existing literature remain very limited. Current investigations are primarily focused on small molecular systems [21,22,23,24,25]. Davy et al. investigated the structures of AlNH_2_, AlNH_3_, and AlNH_4_ clusters using ab initio molecular electronic structure methods [22], finding that the number of hydrogen atoms on the aluminum atom significantly modulates its Lewis acidity, thereby influencing the strength of the Al–N bond. Furthermore, the structures and reaction energies of cyclic and cage-like aluminum–nitrogen–hydrogen clusters have also attracted attention [23]. For instance, a comparison between the (HAl–NH)_2_ and (H_2_Al–NH_2_)_2_ tetrameric ring structures revealed that the transition from a saturated to an unsaturated ring requires higher energy, indicating that coordination-saturated AlNH clusters possess greater stability. While these studies provide a preliminary exploration of AlNH cluster systems, there is still no systematic report on how to precisely regulate their geometric structures and electronic properties through hydrogen atoms.

Previous studies have reported a fully hydrogenated Al_6_N_6_H_12_ [25] cluster that formally achieves tetracoordinate saturation by introducing one H atom to each Al and N atom of the pristine Al_6_N_6_. Nevertheless, this structure merely supplements the coordination number through the formation of Al–H and N–H bonds without altering the symmetry and hexagonal prism geometric framework of the Al_6_N_6_ core. Essentially, it is merely a “hydrogenated derivative” of the original structure, failing to induce substantial geometric reconstruction. This naturally leads to the following question: can hydrogenation be rationally designed to effectively modulate both the geometry and electronic properties of AlN clusters?

To address this, we propose a “selective hydrogenation” strategy for structural modification and property tuning of AlN clusters. Using the smallest three-dimensional Al*_n_*N*_n_*-type cluster, Al_6_N_6_ (**0**), as the precursor, the stepwise introduction of eight H atoms transforms the bilayer hexagonal prism into a cuboid-shaped Al_6_N_6_H_8_ cluster (**1**), accompanied by pronounced charge redistribution and chemical-bonding rearrangements. Notably, its HOMO–LUMO gap increases significantly, reflecting enhanced electronic stability.

This work thus demonstrates that targeted hydrogenation can fundamentally reshape the structures and properties of AlN clusters. The strategy not only enables precise tuning of geometry and stability but also provides reactive hydrogen sites in the newly formed surface Al–H/N–H bonds, laying the foundation for further functionalization and potential applications of the cluster.

## 2. Results and Discussion

### 2.1. Designing the Al_6_N_6_H_8_ Cluster

**Figure 1:**
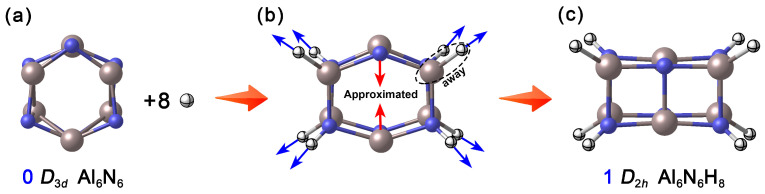
Basic idea of designing Al_6_N_6_H_8_ (**1**) clusters. (**a**) Reported neutral Al_6_N_6_ cluster; (**b**) The trend of change when introducing eight hydrogen atoms; (**c**) Optimized Al_6_N_6_H_8_ cluster. The N atoms are shown in blue, Al in light pink, and H in white.

In nature, aluminum (Al) and nitrogen (N) tend to form tetrahedrally coordinated configurations, as exemplified by the wurtzite-type crystal structure of aluminum nitride (AlN). However, the neutral Al_6_N_6_ (**0**, *D*_3*d*_) [7,15] (Figure 1a) reported in existing theoretical studies adopts a hexagonal prism structure composed of two layers of six-membered rings, where each Al and N atom is tricoordinate. This low-coordination unsaturated state not only endows the cluster with high chemical reactivity but also provides room for its structural regulation and modification. Based on this, the present study proposes a “selective hydrogenation” modification strategy. How were the specific hydrogenation sites determined? To address this question, we systematically calculated the relative energies of various possible structures formed during the stepwise hydrogenation of Al_6_N_6_ (**0**). As shown in Figure S1, upon the introduction of the first hydrogen atom, hydrogen preferentially binds to a nitrogen atom. The structure with hydrogen attached to nitrogen is approximately 34.3 kcal·mol^−1^ lower in energy than the configuration where hydrogen is bonded to an aluminum atom. When adding the second hydrogen atom, it tends to bond to the aluminum atom located on the same side as the initially hydrogenated nitrogen atom. Calculation results indicate that the third and fourth hydrogen atoms preferentially occupy the para-positioned nitrogen and aluminum atoms, respectively. In general, hydrogen atoms added in odd-numbered steps exhibit a stronger tendency to bond with nitrogen atoms, while those added in even-numbered steps favor attachment to aluminum atoms situated on the same side as previously hydrogenated nitrogen atoms. When eight hydrogen atoms are introduced, the four Al atoms and four N atoms on two opposite quadrangular side faces of Al_6_N_6_ (**0**) are hydrogenated, constructing the Al_6_N_6_H_8_ cluster. Interestingly, during the geometry optimization process, the hydrogenation sites are stretched outward due to the formation of Al–H and N–H covalent bonds (4 each), leading to the distortion of the hexagonal prism framework. Meanwhile, the unhydrogenated, para-positioned Al and N atoms on the other two sides approach each other under the transfer of skeletal tension, ultimately forming a new set of Al–N single bonds in the upper and lower layers, respectively. As shown Figure 1, this synergistic rearrangement drives a fundamental transformation of the cluster structure: from the pristine honeycomb-like hexagonal prism to a cuboid configuration. As a result, all Al and N atoms achieve tetracoordinate saturation, and the overall shape and physicochemical properties of the cluster are significantly altered.

**Figure 2:**
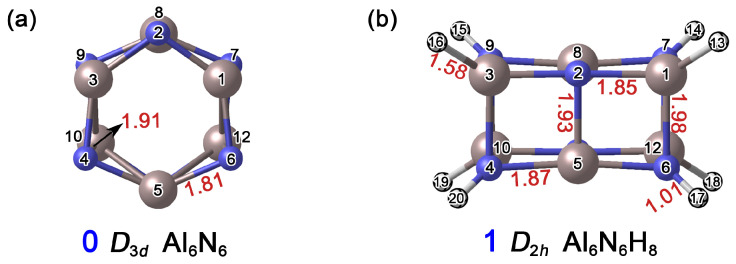
Optimized structures of (**a**) Al_6_N_6_ (**0**) and (**b**) Al_6_N_6_H_8_ (**1**) at the PBE0/def2-TZVPP level. The interatomic distances are given in Å (red color). The atomic numbers are displayed in black font. The N atoms are shown in blue, Al in light pink, and H in white.

As shown in Figure 2, the distances between Al1–N2 and Al1–N7 in the Al_6_N_6_ (**0**) cluster are 1.81 Å and 1.91 Å, respectively. In cluster Al_6_N_6_H_8_ (**1**), the corresponding Al1–N2, Al1–N6, N4–Al5, and Al2–N5 bond lengths range from 1.85 Å to 1.98 Å. Based on the covalent radii recommended by Pyykkö [26], the upper limits for single-bond lengths of Al–N, Al–H, and N–H are 1.97 Å, 1.58 Å, and 1.03 Å, respectively, while the Al=N double bond is approximately 1.63 Å in length. Accordingly, all Al–N bonds in **0** can be preliminarily identified as single bonds. Similarly, the Al–N, Al–H, and N–H interactions in **1** are consistent with a single-bond character. Notably, the Al1–N6 distance in **1** is 1.98 Å, which slightly exceeds the covalent single-bond limit. This slight elongation can be attributed to the formation of new chemical bonds following hydrogen atom addition, where both steric hindrance and charge redistribution collectively lead to an outward displacement of the atoms.

### 2.2. Structures and Stability

**Figure 3:**
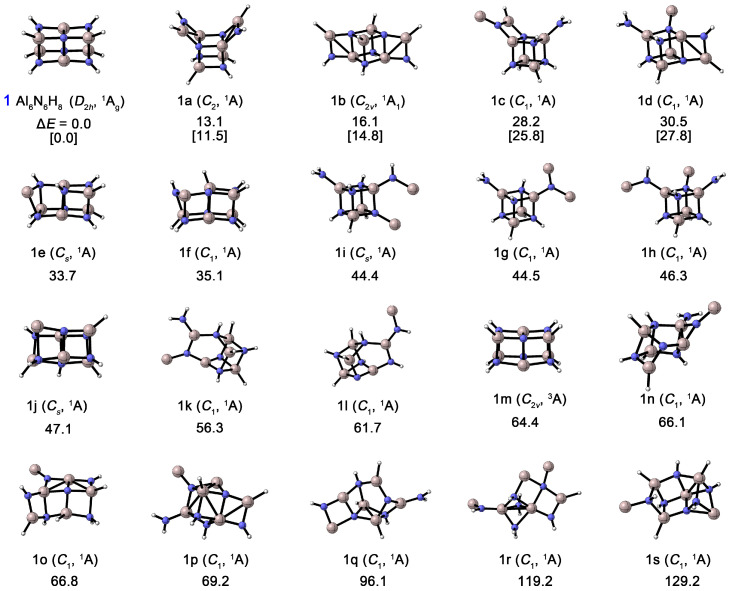
Optimized global-minimum structures of Al_6_N_6_H_8_ (**1**) and the nineteen lowest-lying isomers (**1a**–**1s**). Relative energies are shown in kcal mol^−1^ at the PBE0/def2-TZVPP level. Also shown are energetics data for the top 5 lowest-energy isomers at the single-point CCSD(T)/def2-TZVPP//PBE0/def2-TZVPP level with zero-point energy (ZPE) corrections at PBE0 (in square brackets).

As shown in Figure 3, the thermodynamic stability of Al_6_N_6_H_8_ (**1**) was compared with that of its nineteen isomers. All structures were confirmed to possess no imaginary frequencies through harmonic vibrational frequency calculations, confirming that each structure corresponds to a true minimum on the potential energy surface. At the CCSD(T)/def2-TZVPP//PBE0/def2-TZVPP level, structure **1** is confirmed to be the global minimum (GM), lying at least 11.5 kcal mol^−1^ lower in energy than all other isomers. Furthermore, the T1 diagnostic values obtained from the CCSD(T) calculations for these isomers range from 0.012 to 0.014, which is well below the commonly adopted threshold of 0.02, indicating that this level of theory is reliable for describing these systems. The results show that the energy ordering remains fully consistent between CCSD(T) and PBE0, and a large energy gap (>10 kcal mol^−1^) exists between the GM structure (**1**) and the next most stable isomer (**1a**). This significant energy difference, together with the consistent energy trend, strongly supports the reliability of our conclusion regarding the most stable structure. Structure **1** possesses *D*_2*h*_ symmetry, exhibiting an elongated rectangular shape, with eight hydrogen atoms bonded at its vertices. Notably, isomers containing the Al_4_N_4_ unit generally exhibit relatively lower energies. For instance, isomer **1a** (*C*_2_) can be viewed as a GM-like structure in which two Al–N bonds on the shorter edge of the parallelepiped are broken, resulting in two AlNH_2_ units positioned on opposite sides of one face of the Al_4_N_4_ cube, resembling an “open box”. Isomer **1b** (*C*_2*v*_) also retains the Al_4_N_4_ core, but the two AlNH_2_ units are arranged on opposite sides along the diagonal of the cube. Isomers **1c**–**1j** likewise feature the central Al_4_N_4_ unit, with the remaining atoms forming various groups (e.g., NH_2_) attached irregularly at the cube vertices. In contrast, structures **1k**–**1s** (except for the triplet state **1m**), in which the Al_4_N_4_ unit is disrupted, are all at least 56.3 kcal mol^−1^ higher in energy than **1**. The GM structure contains two coupled Al_4_N_4_ units, and its excellent thermodynamic stability suggests a high potential for formation and characterization in gas-phase experiments.

**Figure 4:**
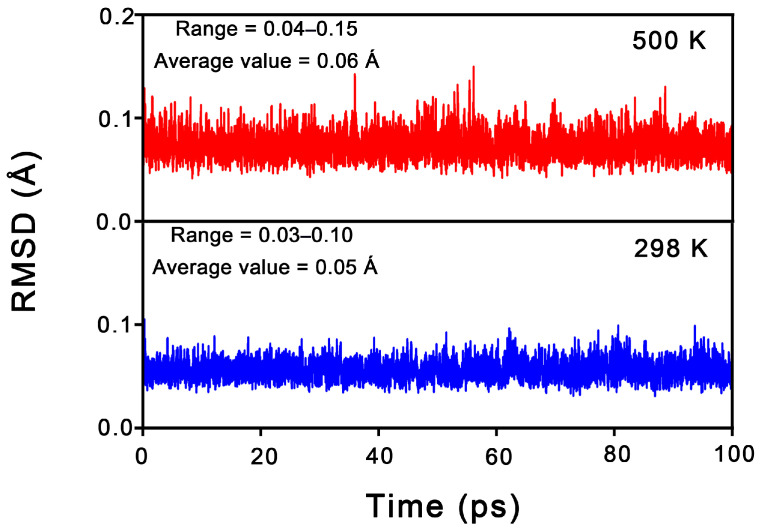
RMSD (in Å) versus simulation time (in ps) for the BOMD simulations of Al_6_N_6_H_8_ (**1**) at the PBE/DZVP level and 298 and 500 K.

From an experimental characterization perspective, the dynamic stability of a cluster is as crucial as its thermodynamic stability. To assess this, Born–Oppenheimer molecular dynamics (BOMD) simulations were conducted for **1** at the PBE/DZVP level, with the time to 100 ps carried out at 298 K and 500 K. The structural evolution was described using the root mean square deviation (RMSD, in Å); smaller RMSD fluctuations correspond to greater dynamic stability. As shown in Figure 4, **1** exhibits relatively excellent dynamic stability at both 298 K and 500 K, maintaining its original framework throughout the simulations. The average RMSD values for **1** at 298 K and 500 K are 0.06 Å and 0.07 Å, respectively. These small values suggest that cluster **1** possesses strong resistance to isomerization and dissociation.

### 2.3. Electronic Structure Analyses

**Figure 5:**
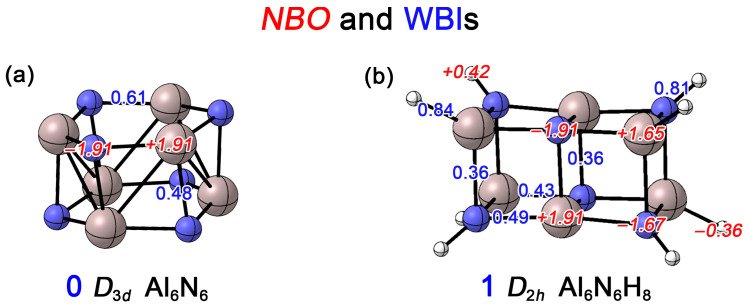
Calculated Wiberg bond indices (WBIs, blue font) and natural atomic charges (in |e|, red font) of (**a**) Al_6_N_6_ (**0**) and (**b**) Al_6_N_6_H_8_ (**1**) at the PBE0/def2-TZVPP level.

In this work, we first conducted Natural Bond Orbital (NBO) analyses to determine the Wiberg bond indices (WBIs) and NBO charges for the Al_6_N_6_ (**0**) and Al_6_N_6_H_8_ (**1**) clusters. As shown in Figure 5a, the WBI for the Al–N bonds within the hexagonal rings of **0** is 0.61, while the WBI for the interlayer Al–N bonds connecting the upper and lower rings is 0.48. Both values are significantly less than one, which is primarily attributed to the substantial electronegativity difference between Al and N, leading to obvious charge transfer. Each N atom carries a charge of −1.91 |e|, whereas each Al atom carries +1.91 |e|. This indicates that the Al–N bonds exhibit not only a covalent character but also a considerable ionic character. In contrast, for **1** (Figure 5b), the WBIs for the Al1–N2 and Al5–N4 bonds are 0.49 and 0.43, respectively. All Al–N bonds on the short edges show a WBI of 0.36, consistent with ionic covalent bonds. Compared to cluster **0**, all Al–N WBIs show a slight decrease. This is attributed to the transformation of the 4c–2e delocalized bond (primarily dominated by the lone pair on the N atoms) into other 2c–2e bonds upon hydrogenation (see the AdNDP analysis below). The N–H and Al–H bonds exhibit WBIs of 0.81 and 0.84, respectively, which are typical for single bonds. Due to the lower electronegativity of Al compared to H, significant charge transfer occurs in the Al–H bonds, where the vertex Al atoms carry a charge of +1.65 |e|, while the bonded H atoms become negatively charged. Similarly, because N is more electronegative than H, the vertex N atoms carry −1.67 |e|. For the Al and N atoms located at the centers of the long edges, the charges are +1.91 |e| and −1.91 |e|, respectively, identical to those in Al_6_N_6_ (**0**). Overall, as shown in Figure S2, a comparison of the electrostatic potential (ESP) maps of the Al_6_N_6_ (**0**) and Al_6_N_6_H_8_ (**1**) clusters reveals that Al_6_N_6_H_8_ (**1**) exhibits a narrower ESP distribution. This indicates that the introduction of hydrogen leads to a more homogeneous charge distribution within the cluster.

**Figure 6:**
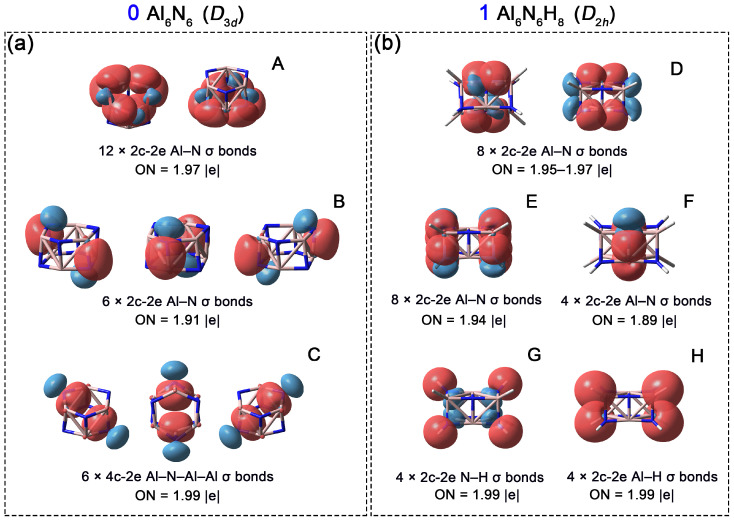
Chemical bonding patterns of Al_6_N_6_ (**0**) and Al_6_N_6_H_8_ (**1**) according to the AdNDP analysis. Occupation numbers (ONs) are denoted. (**a**) (**A**) Twelve 2c-2e Al–N σ bonds along the six edges of each hexagon; (**B**) Six 2c-2e Al–N σ bonds connect the two layered hexagons; (**C**) Six 4c-2e Al–N–Al–Al delocalized σ bonds; (**b**) (**D**) Eight 2c-2e Al–N σ bonds along the long edges of the cuboid; (**E**) Eight 2c-2e Al–N σ bonds are located on the short edges; (**F**) Four Al–N σ bonds in the central region of the cuboid; (**G**) Four N–H σ bonds. (**H**) Four Al–H σ bonds.

Subsequently, to compare chemical bonding in **0** and **1**, Adaptive Natural Density Partitioning (AdNDP) analyses were performed. As shown in Figure 6a, the Al_6_N_6_ cluster (**0**) possesses 24 pairs of valence electrons. The analysis reveals twelve 2c-2e Al–N σ bonds along the six edges of each hexagon with occupation numbers (ONs) of 1.97 |e| (orbital A). Additionally, six 2c-2e Al–N σ bonds (orbital B, ON = 1.91 ∣e∣) connect the two layered hexagons. The remaining six pairs of valence electrons correspond to 4c-2e delocalized σ bonds (orbital C, ON = 1.99 ∣e∣), each involving one N atom and three Al atoms. An alternative interpretation of orbital C is to view it as six lone pairs on the nitrogen atoms, with an ON of 1.84 |e| (Figure S1). Concurrently, the ONs of the Al–N 2c-2e bonds decrease to 1.94 and 1.88 |e|, respectively. This scheme further explain that the primary contribution to the 4c-2e bond originates from N lone pairs. After the addition of eight hydrogen atoms, the system gains eight electrons (one from each H atom), resulting in a total of 28 valence electron pairs for Al_6_N_6_H_8_ (**1**). As shown in Figure 6b, the AdNDP scheme identifies eight 2c-2e Al–N σ bonds along the long edges of the cuboid (orbital D, ON = 1.95–1.97 ∣e∣). Another eight 2c-2e Al–N σ bonds are located on the short edges (orbital E, ON = 1.94 ∣e∣). In contrast to **0**, four additional Al–N σ bonds are observed in the central region of the cuboid in **1** (orbital F, ON = 1.89 ∣e∣). Overall, **1** features two more Al–N bonds than **0**. The formation of these new Al–N σ bonds is attributed to the introduction of eight hydrogen atoms at the vertices of **0**: the repulsive forces induced by hydrogenation elongate the original double-layered hexagonal structure, causing the Al and N atoms along the central diagonal to approach each other passively, thereby promoting the formation of these new Al–N bonds. The remaining eight pairs of valence electrons correspond to four Al–H bonds and four N–H bonds (2c-2e each).

**Figure 7:**
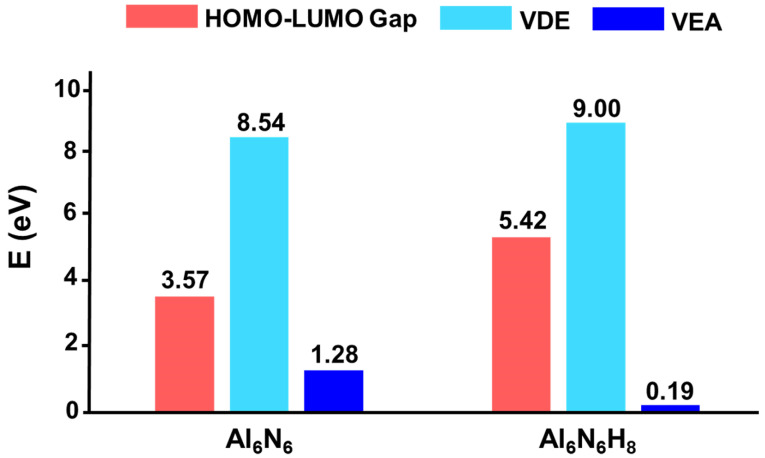
Comparison of HOMO–LUMO gaps (in eV) between Al_6_N_6_ (**0**) and Al_6_N_6_H_8_ (**1**) at the PBE0/def2-TZVPP level.

The HOMO–LUMO gap serves as a critical indicator of a cluster’s electronic stability, revealing its propensity to participate in chemical reactions. Generally, a larger HOMO–LUMO gap correlates with higher chemical stability. As shown in Figure 7, the HOMO–LUMO gap of Al_6_N_6_ (**0**) is 3.57 eV. Interestingly, hydrogenation leads to a significant enhancement in electronic stability, with the gap increasing to 5.42 eV for Al_6_N_6_H_8_ (**1**), which is 1.85 eV higher than that of **0**. This indicates that hydrogen incorporation effectively modulates the electronic structure, endowing **1** with considerably improved electronic stability. Furthermore, the vertical detachment energy (VDE) and vertical electron affinity (VEA) of both clusters were calculated at the OVGF/def2-TZVPP level. For a neutral cluster, a higher VDE value reflects greater resistance to electron loss, while a lower VEA value suggests a reduced tendency to accept an additional electron. The computed VDE of **1** is 9.00 eV, which is higher than that of **0** (8.54 eV), while its VEA is 0.19 eV, which is substantially lower than the 1.28 eV of **0**. These results indicate that hydrogenation renders cluster **1** more resistant to both electron loss and electron capture, confirming that its electronic structure is more robust than that of its precursor.

### 2.4. Functionalization Regulation

**Figure 8:**
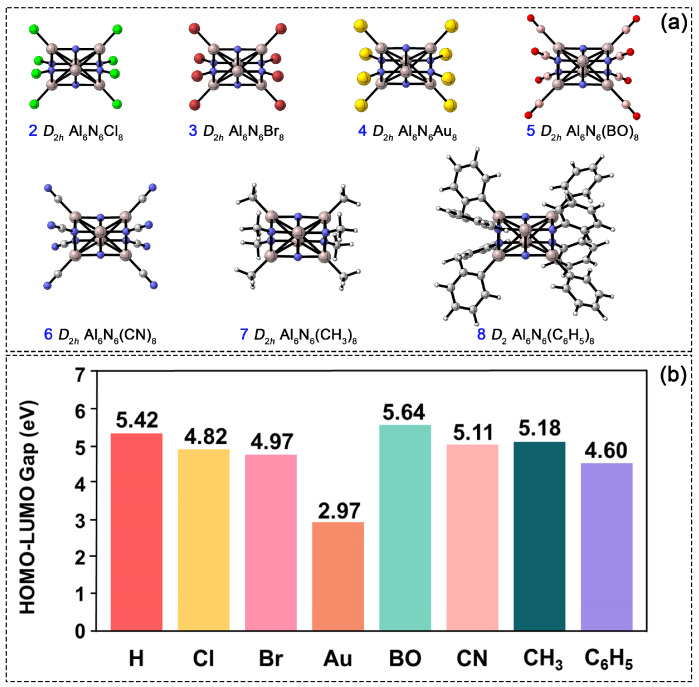
(**a**) PBE0/def2-TZVPP-optimized structures of Al_6_N_6_E_8_ (E = Cl, Br, Au, BO, CN, CH_3_, C_6_H_5_, **2**–**8**). (**b**) Comparison of the HOMO-LUMO gap among **2**–**8**.

In the Al_6_N_6_H_8_ (**1**) cluster, the hydrogen atom’s positions serve as substitutable sites, enabling the modulation of the cluster’s structure and properties. To investigate the influence of diverse substituents on electronic stability, we systematically replaced all hydrogen atoms with seven different species, as shown in Figure 8a. The substituents strategically selected in this work cover a diverse range of electronic and steric properties, including halogens (Cl and Br) as electronegative elements with strong electron-withdrawing properties, Au as a transition metal atom with potential catalytic applications, boronyl (BO) [27] as an emerging novel ligand with prominent electron-withdrawing characteristics, cyano (CN) and methyl (CH_3_) as typical common functional groups in organic chemistry, and phenyl (C_6_H_5_) as a sterically bulky aromatic group for investigating steric hindrance effects. The following discussion focuses on these fully octa-substituted derivatives. Computational results indicate that at the PBE0/def2-TZVPP level, structures **2**–**7** retain *D*_2_*_h_* symmetry and are confirmed by frequency calculations to possess no imaginary frequencies, with each corresponding to a true minimum on the potential energy surface. In contrast, when optimized under *D*_2_*_h_* symmetry constraints, structure **8** exhibits an imaginary frequency, primarily due to the steric hindrance of the phenyl substituent. After relaxation along the imaginary vibrational mode, the structure stabilizes into a lower *D*_2_ symmetry. The corresponding atomic displacements for imaginary frequency vibrational modes under *D*_2_*_h_* symmetry are provided in Figure S4. The bond lengths of Al1–N2, Al1–N6, N4–Al5, and Al2–N5 in structures **2**–**8** range from 1.84 to 1.86 Å, from 1.97 to 2.00 Å, from 1.87 to 1.89 Å, and from 1.91 to 1.93 Å, respectively. These values closely match the corresponding bond lengths in **1**, indicating that the cuboid Al_6_N_6_ core’s framework remains stable upon complete hydrogen substitution. However, the electronic properties of **2**–**8** differ noticeably from those of the parent cluster (**1**). As shown in Figure 8b, all atom-substituted clusters (2–4) exhibit a narrower HOMO–LUMO gap compared to **1**. Among them, the Au-substituted structure shows the smallest gap, suggesting its higher chemical reactivity. In contrast, substitution with the BO group results in the widest HOMO–LUMO gap: 0.22 eV larger than that of **1** and exceeding those of the organic-group-substituted clusters (CN, CH_3_, and C_6_H_5_) by 0.53, 0.46, and 1.04 eV, respectively. Thus, the BO-substituted derivative possesses the most stable electronic structure among all substituted systems studied.

### 2.5. Simulated IR Spectrums

**Figure 9:**
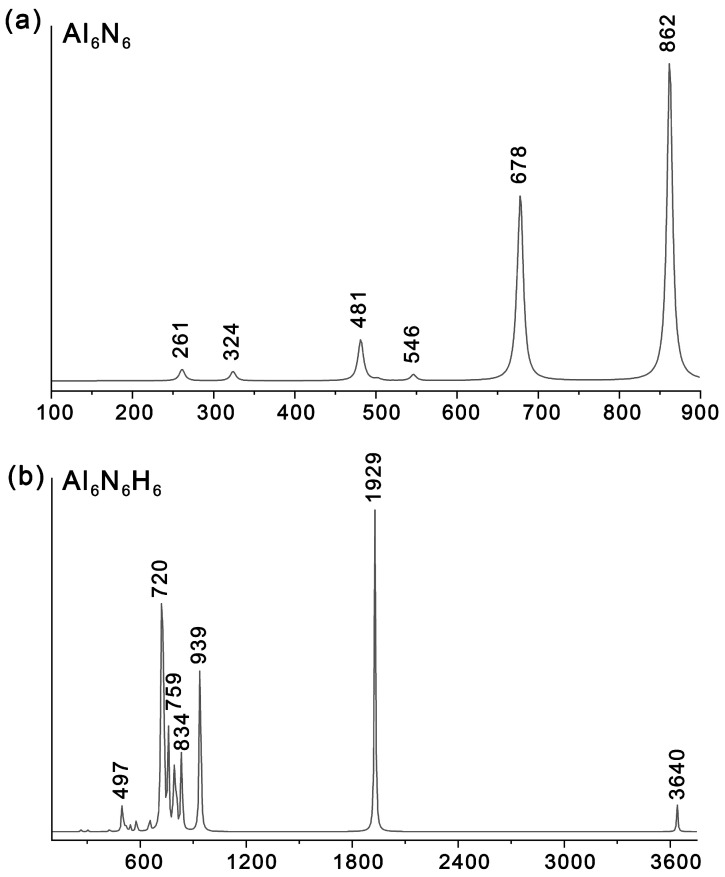
Simulated IR spectra of (**a**) Al_6_N_6_ and (**b**) Al_6_N_6_H_8_ (**1**) at the PBE0/def2-TZVPP level.

To facilitate future experimental characterization, we simulated the infrared (IR) spectra of Al_6_N_6_ (**0**) and Al_6_N_6_H_8_ (**1**) (Figure 9) at the PBE0/def2-TZVPP level and compared them with those of the precursor structure, Al_6_N_6_ (**0**). As shown in Table 1, the key information on the main vibrational peaks for **0** and **1** is detailed, covering peak positions, activity intensities, vibrational mode types, and specific vibrational assignment. For **1**, the most intense IR absorption peak is observed at 1929 cm^−1^, which is primarily attributed to the stretching vibration of the Al–H bonds at the vertices. A weak peak at 497 cm^−1^ is mainly ascribed to the bending vibration of Al–H bonds. The weak feature at 3640 cm^−1^ corresponds to N–H stretching vibrations, while the signals spanning 759–834 cm^−1^ correspond to N–H bending modes. Notably, the second most intense peak is located at 720 cm^−1^, corresponding to Al–N bending vibrations, and the third most intense peak at 939 cm^−1^ is assigned to Al–N stretching vibrations. In contrast, the IR spectrum of **0** exhibits characteristic Al–N bending and stretching vibrations at 678 cm^−1^ and 862 cm^−1^, respectively. And the corresponding atomic displacement vectors of the IR vibrational mode of both Al_6_N_6_ (**0**) and Al_6_N_6_H_8_ (**1**) are shown in Table S2. In addition, it should be noted that introducing eight hydrogen atoms induces a distinct blue shift in these characteristic Al–N stretching and bending vibrational modes.

## 3. Methods

The geometry optimization and harmonic vibrational frequency analyses of the pristine honeycomb-like hexagonal prism Al_6_N_6_ cluster and the newly designed Al_6_N_6_H_8_ cluster were performed at the PBE0/def2-TZVPP [28,29] level. The potential energy surface (PES) calculations for Al_6_N_6_H_8_ were carried out using the Coalescence-Kick (CK) [30,31] and Basin-Hopping algorithms [32]; both singlet and triplet states were considered, and the initial structures were first generated and optimized at the PBE/DZVP level. Approximately 3000 stationary points were probed for Al_6_N_6_H_8_. Twenty low-energy isomers were then reoptimized at the PBE0/def2-TZVPP level. Among these, the relative single-point energies of the top five structures were further evaluated at the CCSD(T)/def2-TZVPP level [33,34] with zero-point energy (ZPE) corrections at the PBE0/def2-TZVPP level [abbreviated as CCSD(T) + ZPE_PBE0_], while the PBE0 functional was used for subsequent electronic structure analyses. Chemical bonding was analyzed using Adaptive Natural Density Partitioning (AdNDP) [35] at the PBE0/def2-TZVPP level. Dynamic stability was assessed via Born–Oppenheimer molecular dynamics (BOMD) simulations at selected temperatures using the CP2K package [36] at the PBE/DZVP level. Vertical detachment energies (VDEs) and vertical electron affinities (VEAs) were calculated using the Outer-Valence Green’s Function (OVGF) method [37]. Natural bond orbital (NBO) analysis was performed using the NBO 6.0 program [38]. AdNDP analysis used Multiwfn 3.8 [39]. The Basin-Hopping algorithm was coded in the TGMin 2.0 program [40]. The CCSD(T) calculations were carried out with MolPro 2012.1 [41], and all other calculations were performed using Gaussian 16 (A.03) [42]. The results were visualized with GaussView 6.1 and CYLview 2.0.

## 4. Conclusions

This study demonstrates that the bilayer honeycomb-like hexagonal prism of Al_6_N_6_ (**0**) can be reconstructed into a thermodynamically and dynamically stable cuboid structure, Al_6_N_6_H_8_ (**1**), through a “selective hydrogenation” strategy. In cluster **1**, the Al–N, Al–H, and N–H bonds are identified as covalent single bonds with an ionic character resulting from the electronegativity differences among Al, N, and H. The HOMO–LUMO gap of **1** increase by 1.85 eV compared to that of **0**, indicating a significant enhancement in electronic stability upon hydrogenation. Substituting the eight hydrogen atoms in **1** with various atoms or groups preserves the cuboidal Al_6_N_6_ core framework while enabling notable modulation of its electronic properties. Furthermore, the simulated infrared spectrum reveals an overall blue shift in the Al–N vibrational modes after hydrogenation, providing a theoretical reference for future experimental characterization. In summary, selective hydrogenation serves as an effective strategy for tuning the structure and physicochemical properties of AlN clusters, offering a feasible pathway for the design of novel clusters.

## Figures and Tables

**Table 1:** All peak positions are theoretical values calculated at the PBE0/def2-TZVPP level.

Molecular System	Calculated Peak Position (cm^−1^)	IR Activity Intensity	Vibrational Mode Type	Specific Vibrational Assignment
**Al_6_N_6_ (0)**	862	Strong (s)	Stretching (ν)	Al–N framework stretching vibration
	678	Medium Strong (ms)	Bending (δ)	Al–N framework bending vibration
**Al_6_N_6_H_8_ (1)**	3640	Weak (w)	Stretching (ν)	N–H bond stretching vibration
	1929	Very Strong (vs)	Stretching (ν)	Terminal Al–H bond stretching vibration
	939	Strong (s)	Stretching (ν)	Al–N framework stretching vibration
	834~759	Weak (w)	Bending (δ)	In-plane N–H bond bending vibration
	720	Medium Strong (ms)	Bending (δ)	Al–N framework bending vibration
	497	Weak (w)	Bending (δ)	In-plane Al–H bond bending vibration

## Data Availability

Data are contained within the article and Supplementary Materials.

## Supplementary Materials

The following supporting information can be downloaded at https://www.mdpi.com/article/10.3390/molecules31030495/s1, Table S1: Cartesian coordinates for optimized global-minimum (GM) structures of Al_6_N_6_ (**0**) and Al_6_N_6_H_8_ (**1**) clusters at the PBE0/def2-TZVPP level; Table S2: Simulated infrared (IR) vibrational mode assignment and atomic displacement maps of Al_6_N_6_ (**0**) and Al_6_N_6_H_8_ (**1**). This table summarizes the calculated IR vibrational peaks, activity intensities, mode types, and corresponding atomic displacement vectors for both the Al_6_N_6_ (**0**) and Al_6_N_6_H_8_ (**1**), at the PBE0/def2-TZVPP level. Figure S1: A structural schematic diagram illustrating the stepwise hydrogenation of Al_6_N_6_ (**0**) at different sites, with energies given in kcal mol^−1^ at the PBE0/def2-TZVPP level. Figure S2: Electrostatic potential (ESP) maps of (A) Al_6_N_6_ (**0**) and (B) Al_6_N_6_H_8_ (**1**). Figure S3: An alternative chemical bonding pattern of Al_6_N_6_ (**0**) according to the AdNDP analysis. Occupation numbers (ONs) are denoted. Figure S4: Optimized equilibrium structures of the *D*_2_ (left) and *D*_2*h*_ (right) isomers of Al_6_N_6_(C_6_H_5_)_8_, with the number of imaginary vibrational frequencies (NImag) indicated. Atomic displacement vectors corresponding to the imaginary vibrational modes of the *D*_2*h*_ isomer, with imaginary frequencies of (a) 43.40i, (b) 30.88i, (c) 28.27i, (d) 20.30i, and (e) 10.32i cm^−1^, respectively.
