# Supplementary material for: Structural and Electronic Stabilization Tuning of Al6N6 Clusters via Hydrogenation: A Theory Study of Al6N6H8
Source: Molecules. 2026 Jan 31;31(3):495. doi: 10.3390/molecules31030495 (PMC12899515; doi:10.3390/molecules31030495)

SUPPLEMENTARY INFORMATION

Structural and Electronic Stabilization Tuning of Al_6N_6 Clusters via Hydrogenation: A Theory Study of $Al_6N_6H_8$

Peng-Fei Li [1,*], Yang Yang [2] and Shu-Juan Gao [3,*]

[1] Institute of Technology, Shanxi Open University, Taiyuan 030027, China
[2] The Key Laboratory of the Materials for Energy Conversion and Storage of Shanxi Province, Institute of Molecular Science, Shanxi University, 92 Wucheng Road, Taiyuan 030006, China; yangyang9@sxu.edu.cn
[3] Department of Chemical and Materials Engineering, Lyuliang University, Lvliang 033001, China
* Correspondence: lipengfei@sxu.edu.cn (P.-F.L.); shujuangao@llu.edu.cn (S.-J.G.)

Table S1. Cartesian coordinates for optimized global-minimum (GM) structures of Al_6N_6 (**0**) and $Al_6N_6H_8$ (**1**) clusters at the PBE0/def2-TZVPP level.

Table S2. Simulated infrared (IR) vibrational mode assignment and atomic displacement maps of Al_6N_6 (**0**) and $Al_6N_6H_8$ (**1**). This table summarizes the calculated IR vibrational peaks, activity intensities, mode types, and corresponding atomic displacement vectors for both the Al_6N_6 (**0**) and $Al_6N_6H_8$ (**1**), at the PBE0/def2-TZVPP level.

Figure S1. A structural schematic diagram illustrating the stepwise hydrogenation of Al_6N_6 (**0**) at different sites, with energies given in kcal mol^{-1} at the PBE0/def2-TZVPP level.

Figure S2. Electrostatic potential (ESP) maps of (A) Al_6N_6 (**0**) and (B) $Al_6N_6H_8$ (**1**).

Figure S3. An alternative chemical bonding pattern of Al_6N_6 (**0**) according to the AdNDP analysis. Occupation numbers (ONs) are denoted.

Figure S4. Optimized equilibrium structures of the D_2 (left) and D_{2h} (right) isomers of $Al_6N_6(C_6H_5)_8$, with the number of imaginary vibrational frequencies (NImag) indicated. Atomic displacement vectors corresponding to the imaginary vibrational modes of the D_{2h} isomer, with imaginary frequencies of (a) 43.40i, (b) 30.88i, (c) 28.27i, (d) 20.30i, and (e) 10.32i cm^{-1}, respectively.

Table S1. Cartesian coordinates for optimized global-minimum (GM) structures of Al_6N_6 (**0**) and $Al_6N_6H_8$ (**1**) clusters at the PBE0/def2-TZVPP level.

GM, Al_6N_6 (**0**, D_{3d})

Al	0.00000000	1.68927000	0.88121600
Al	-1.46295000	0.84463500	-0.88121600
Al	-1.46295000	-0.84463500	0.88121600
Al	1.46295000	0.84463500	-0.88121600
Al	1.46295000	-0.84463500	0.88121600
Al	0.00000000	-1.68927000	-0.88121600
N	-1.64968400	-0.95244600	-1.02122500
N	0.00000000	1.90489100	-1.02122500
N	1.64968400	-0.95244600	-1.02122500
N	1.64968400	0.95244600	1.02122500
N	-1.64968400	0.95244600	1.02122500
N	0.00000000	-1.90489100	1.02122500

GM, $Al_6N_6H_8$ (**1**, D_{2h})

Al	1.85091300	1.39193400	0.00000000
Al	1.85091300	-1.39193400	0.00000000
Al	0.00000000	0.00000000	1.28138500
Al	0.00000000	0.00000000	-1.28138500
Al	-1.85091300	1.39193400	0.00000000
Al	-1.85091300	-1.39193400	0.00000000
N	0.00000000	-1.43746200	0.00000000
N	1.86477500	0.00000000	-1.40181300

N	-1.86477500	0.00000000	-1.40181300
N	0.00000000	1.43746200	0.00000000
N	1.86477500	0.00000000	1.40181300
N	-1.86477500	0.00000000	1.40181300
H	2.91953900	2.55684800	0.00000000
H	2.51661400	0.00000000	-2.17105400
H	2.91953900	-2.55684800	0.00000000
H	2.51661400	0.00000000	2.17105400
H	-2.91953900	2.55684800	0.00000000
H	-2.51661400	0.00000000	2.17105400
H	-2.51661400	0.00000000	-2.17105400
H	-2.91953900	-2.55684800	0.00000000

Table S2. Simulated infrared (IR) vibrational mode assignment and atomic displacement maps of Al_6N_6 (**0**) and $Al_6N_6H_8$ (**1**). This table summarizes the calculated IR vibrational peaks, activity intensities, mode types, and corresponding atomic displacement vectors for both the Al_6N_6 (**0**) and $Al_6N_6H_8$ (**1**), at the PBE0/def2-TZVPP level.

Molecular System	Calculated Peak Position (cm^{-1})	IR Activity Intensity	Vibrational Mode Type	Atomic displacement map
**Al_6N_6 (0)**	862	Strong (s)	Stretching (ν)	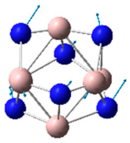
	678	Medium Strong (ms)	Bending (δ)	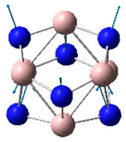
**$Al_6N_6H_8$ (1)**	3640	Weak (w)	Stretching (ν)	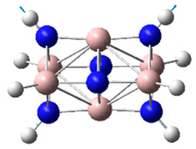
	1929	Very Strong (vs)	Stretching (ν)	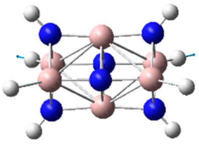
	939	Strong (s)	Stretching (ν)	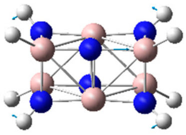
	834~759	Weak (w)	Bending (δ)	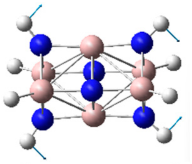 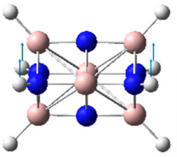

	720	Medium Strong (ms)	Bending (ð)	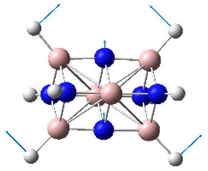
	497	Weak (w)	Bending (ð)	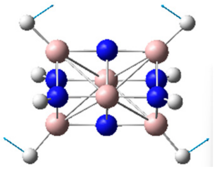

Figure S1. A structural schematic diagram illustrating the stepwise hydrogenation of Al_6N_6 (**0**) at different sites, with energies given in kcal mol^{-1} at the PBE0/def2-TZVPP level.

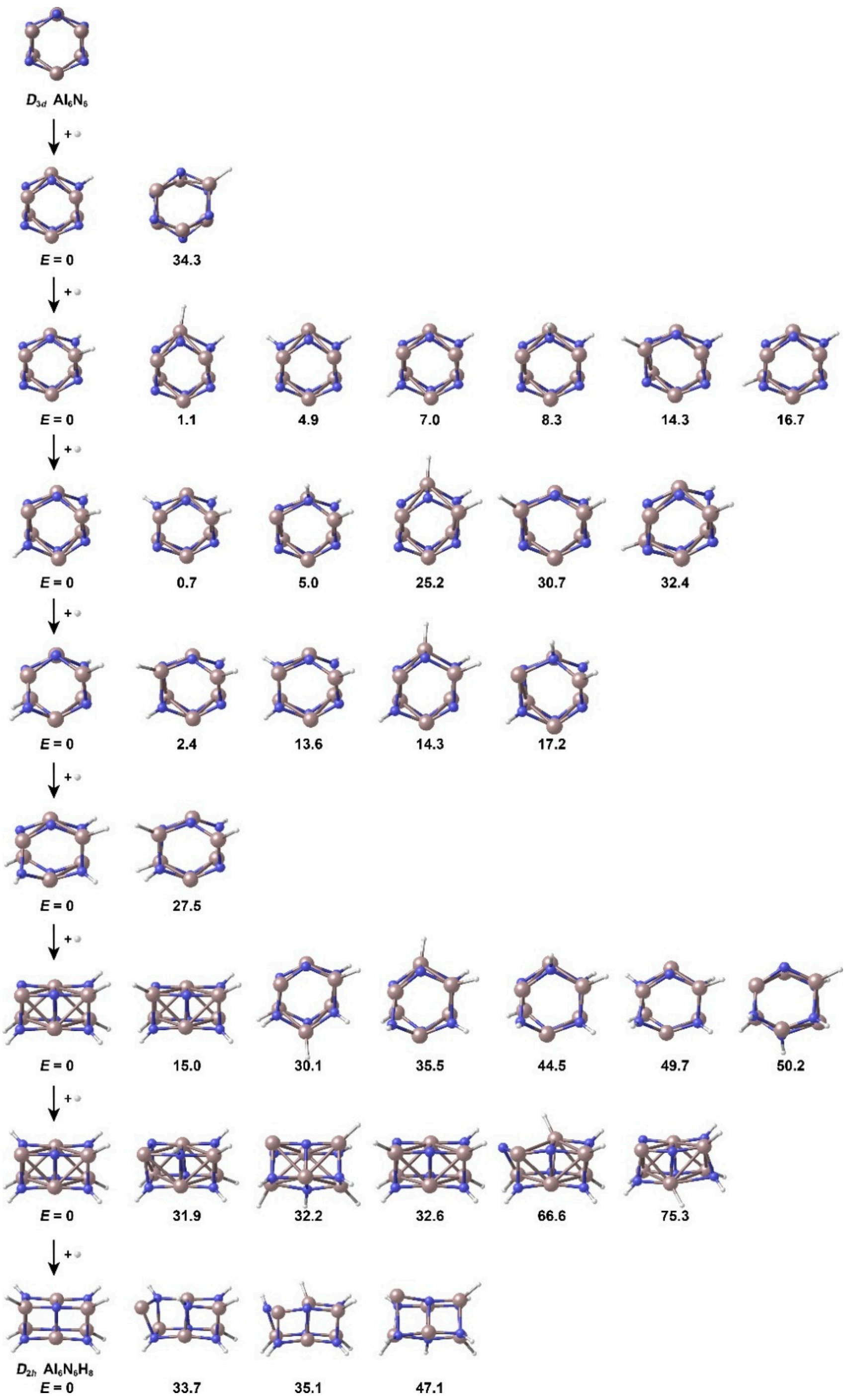

Figure S2. Electrostatic potential (ESP) maps of (A) Al_6N_6 (**0**) and (B) $Al_6N_6H_8$ (**1**).

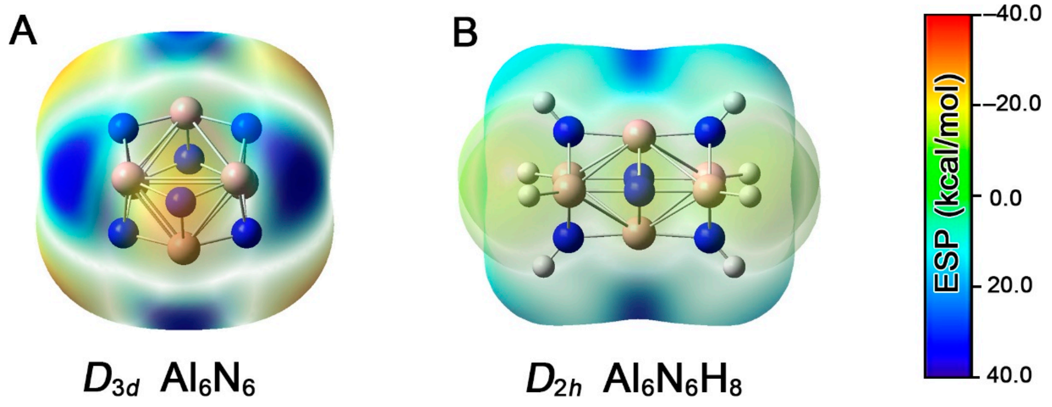

Figure S3. An alternative chemical bonding pattern of Al_6N_6 (**0**) according to the AdNDP analysis. Occupation numbers (ONs) are denoted.

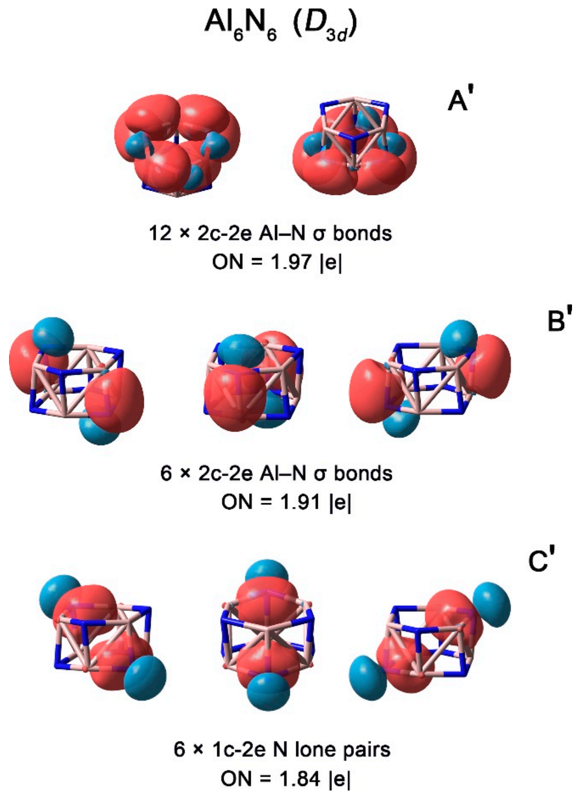

Figure S4. Optimized equilibrium structures of the D_2 (left) and D_{2h} (right) isomers of $Al_6N_6(C_6H_5)_8$, with the number of imaginary vibrational frequencies (NImag) indicated. Atomic displacement vectors corresponding to the imaginary vibrational modes of the D_{2h} isomer, with imaginary frequencies of (a) 43.40i, (b) 30.88i, (c) 28.27i, (d) 20.30i, and (e) 10.32i cm^{-1}, respectively.

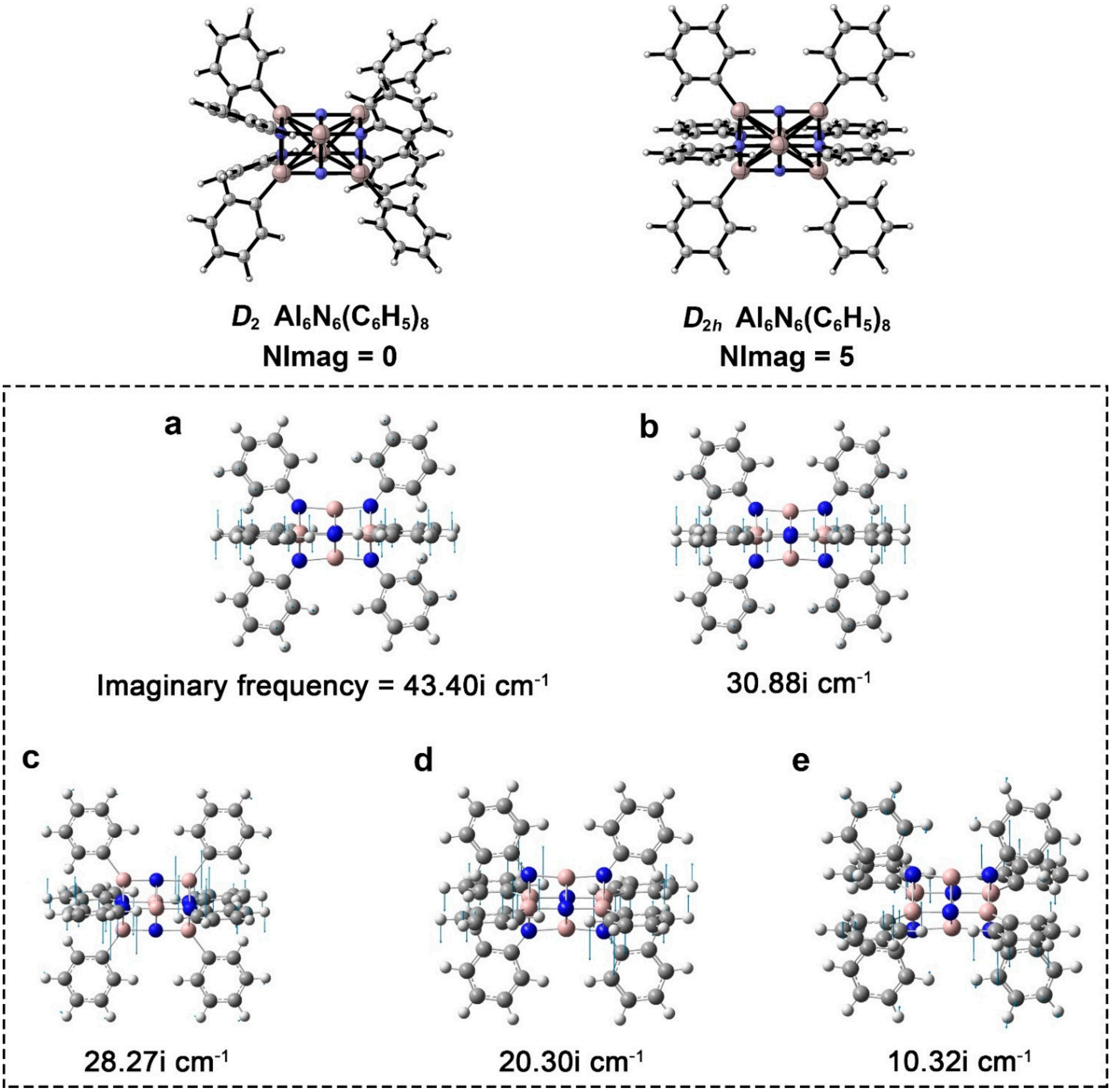

Supplement: Supplementary file 1 [file molecules-31-00495-s001.zip › molecules-4106850-supplementary.pdf]
